# Double Resection of the Lower Maxilla for Protruding Lower Jaw

**Published:** 1899-05

**Authors:** Eugene S. Talbot

**Affiliations:** Chicago, Ill.


### Domestic Correspondence.

### DOUBLE RESECTION OF THE LOWER MAXILLA FOR PROTRUDING LOWER JAW.

To the Editor:

Sir,—My article under this title (International Dental Journal, October, 1898), in the opinion of some critics, seems to require explanation. As a scientific criticism, it was not assumed that it would create the bad feeling evinced in the acrid, shallow criticism it has provoked.

This operation was suggested to me by the late Dr. W. W. Allport a quarter of a century ago, with whom I discussed its merits many times. It seemed to be thoroughly indicated in extreme development of the lower jaw. With Drs. Allport, Brainard, Gunn, and other surgeons, I was of opinion that such an operation was not indicated by the fact that the pulps in the anterior teeth were liable to be destroyed. I have, therefore, refused to attempt the operation for this reason, although I have had a number of patients on whom such an operation might be performed with *éclat.*

The article published by Dr. James W. Whipple in the *Dental Cosmos* for July, 1898, would have passed without notice. I should have waited with great interest for the results of the operation had it not been for the article by Dr. Edward H. Angle (*Dental Cosmos,* August, 1898), in which he claims (page 635) that the " writer was the first to suggest this operation, and has for the past four years been discussing with surgeons its prognosis," etc. In a foot-note it is stated, " Supplement to the Angle System of Regulating and Retention of the Teeth and Fracture of the Maxilla. Now in press." Then follow the photograph of a man and a picture of the relation of the jaws to each other, and a diagram of the proposed operation.

My aim in criticising was dictated by what are usually regarded as the highest motives,—the aim to secure truth, both as to priority and to prevent suffering from premature adoption of an operation which indications (other than commercial advertising reasons) did not justify. The last criticism was doubly justified by the fact that it was proposed to advocate the operation the value of which was not demonstrated in an authoritative text-book.

Illustrations are supposed to illustrate actual conditions. If they are merely hypothetic, the fact should be stated; otherwise their use doubly justifies criticism based on their alleged value as illustrating facts. If an error be dangerous when supposed to be a fact, it is doubly dangerous when the alleged fact turns out to be a mere theory. The intent of the criticisms and their motive were fully explained in a private letter to Dr. Angle, so that there should be no possible misunderstanding.

Certainly this procedure was calculated to destroy any belief in a personal animus, and to show that benefit, not injury, was intended by criticisms which any scientific dentist has the right to make.

I congratulate Dr. James W. Whipple on the (to me) unexpectedly valuable results he has obtained from the operations (*Dental Cosmos,* March, 1899).

If after a series of similar operations like results be obtained, this operation must be recognized by surgeons as one of the most remarkable in modern times, and those who were instrumental in bringing it into use will undoubtedly deserve the highest credit.

Those who are acquainted with me know well that for the past two decades I have emphatically advocated original research. No one in the profession would go further or do more to foster this work than

EUGENE S. TALBOT.

CHICAGO, ILL.

